# Asymmetry in Family History Implicates Nonstandard Genetic Mechanisms: Application to the Genetics of Breast Cancer

**DOI:** 10.1371/journal.pgen.1004174

**Published:** 2014-03-20

**Authors:** Clarice R. Weinberg, Min Shi, Lisa A. DeRoo, Jack A. Taylor, Dale P. Sandler, David M. Umbach

**Affiliations:** 1 Biostatistics Branch, NIEHS, NIH, DHHS, Research Triangle Park, North Carolina, United States of America; 2 Epidemiology Branch, NIEHS, NIH, DHHS, Research Triangle Park, North Carolina, United States of America; University of Alabama at Birmingham, United States of America

## Abstract

Genome-wide association studies typically target inherited autosomal variants, but less studied genetic mechanisms can play a role in complex disease. Sex-linked variants aside, three genetic phenomena can induce differential risk in maternal versus paternal lineages of affected individuals: 1. maternal effects, reflecting the maternal genome's influence on prenatal development; 2. mitochondrial variants, which are inherited maternally; 3. autosomal genes, whose effects depend on parent of origin. We algebraically show that small asymmetries in family histories of affected individuals may reflect much larger genetic risks acting via those mechanisms. We apply these ideas to a study of sisters of women with breast cancer. Among 5,091 distinct families of women reporting that exactly one grandmother had breast cancer, risk was skewed toward maternal grandmothers (p<0.0001), especially if the granddaughter was diagnosed between age 45 and 54. Maternal genetic effects, mitochondrial variants, or variant genes with parent-of-origin effects may influence risk of perimenopausal breast cancer.

## Author Summary

Genetic studies often collect family histories from diagnosed individuals. Some diseases exhibit *inter-lineage asymmetry*: mothers and their progenitors have higher (or lower) risk than fathers and their progenitors, and descendants of female cases have higher (or lower) risk than descendants of male cases. We describe how certain non-standard genetic mechanisms might underlie that asymmetry and make substantial contributions to disease susceptibility. Besides variants on sex chromosomes, these mechanisms include variants in the mother's genome that influence fetal development and hence later risk, variants in the mitochondria that modulate risk, and susceptibility variants in particular inherited genes whose expression depends on whether the variant came from the mother or the father. Applying our ideas to a study of more than 30,000 families with breast cancer, we found that more maternal grandmothers of cases than paternal grandmothers of cases had breast cancer, giving evidence that such non-standard mechanisms may be important contributors to breast cancer risk.

## Introduction

Genome-wide association studies (GWASs) that compare affected individuals and controls have identified many inherited genetic variants associated with complex diseases [1]. Nevertheless, effects of single nucleotide polymorphisms (SNPs) tend to be small [2] and much of the heritability for major diseases remains unexplained. For example, the most important GWAS-derived SNPs for breast cancer explain little of the risk [3].

Four other genetic mechanisms (henceforth referred to as “nonstandard”) are overlooked in a typical GWAS. Sex-linked genetic variants on the Y or the X chromosome are often not considered, although polymorphic X loci may be relevant to breast cancer [4].

The mother's genome can also exert effects on the developing fetus, of consequence for both birth outcomes and adult phenotypes [5], [6], [7]. Such maternally-mediated prenatal effects remain unexplored for breast cancer, though a prenatal influence on adult risk is suggested by the fact that birth weight is a risk factor [8]. A third mechanism involves variants in mitochondrial DNA, as reported for breast cancer in African-American women [9]. Finally, parent-of-origin effects (e.g. due to imprinted polymorphic autosomal genes) can also influence risk, as exemplified by a report based on Icelandic families [10], where the effect of an allele related to breast cancer differed depending on whether its origin was maternal or paternal.

Each of these nonstandard mechanisms produces asymmetry in family history data. We define *inter-lineage asymmetry* as the presence of a higher (or lower) risk either for the mother and her progenitors compared to the father and his progenitors, or for descendants of female cases compared to descendants of male cases. Although Table 1 includes sex-linked effects, we will not consider them further here. A maternally-mediated prenatal effect should produce increased risk in an affected individual's mother's (but not father's) progenitors, in a pattern where risk diminishes toward earlier generations. By contrast, a parent-of-origin effect could show a diminishing pattern of increased risk in the affected individual's *father's* progenitors if only the paternally inherited copy is expressed. The action of these understudied mechanisms can be discerned by studying family histories.

**Table 1:** Qualitative asymmetries produced by non-autosomal genetic mechanisms.

Source of Effect	Index case	Excess risk produced in		Attenuation across generations
		Progenitors	Progeny	
Sex-linked (X)	Male	Maternal but not paternal lineage	Daughters and their progeny but not sons and their progeny	Yes
Sex-linked (X)	Female	Depends on genetic risk model.	Depends on genetic risk model.	Yes
Sex-linked (Y)	Male	Only male-to-male paternal progenitors	Male progeny only	No
Maternally-mediated prenatal effect (autosomal)	Male/female same pattern	Maternal lineages	Offspring of female progeny	Yes
Mitochondria	Female	Maternal lineages	Male and female progeny linked to index case by females	No
Mitochondria	Male	Maternal lineages	No progeny	No
Parent-of-origin effect (autosomal)	Male/female same pattern	Maternal or paternal lineages, depending on which parental allele is expressed	Only progeny of male cases or only progeny of female cases	Yes

The presence of disease in a proband statistically induces enrichment in their progenitors and progeny for risk-related alleles. For the mechanisms in Table 1, that enrichment manifests as inter-lineage asymmetry. To quantify that asymmetry, we define several inter-lineage relative risks, whose exact definitions (and magnitudes) depend on the familial relationship to the affected proband of the individuals whose risks are being compared. We denote mother, father and child by M, F, and C, and extend the same notation for progenitors, e.g., as MM for the mother's mother and MMF for the mother's mother's father. C will denote the grandchild when considering parents of parents. Let 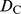, 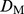, 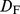 denote the events that the child, mother, father has the disease, respectively, with analogous notation for other relatives. Let 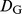, 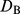 denote the events that a female (girl), male (boy) in the population has the disease, respectively. We can compare risk in the proband's grandparental generation directly by comparing two sex-matched grandparents, either grandmothers (MM vs. FM as 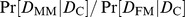) or grandfathers (MF vs. FF as 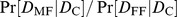), without normalizing to the population. By contrast, assessing asymmetry in risk between mothers and fathers of affected individuals requires normalizing those risks to risk for females versus males in the general population. Thus, the inter-lineage parent relative risk is the risk for the proband's mother compared to that for females in the population divided by the risk for the proband's father compared to that for males in the population, expressed symbolically as 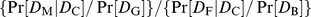.

Investigators have also looked *prospectively* for asymmetry, by comparing risk in the offspring of male versus female affected individuals [11]. The inter-lineage son (daughter) relative risk is the risk for sons (daughters) of affected mothers divided by the risk for sons (daughters) of affected fathers. Let 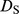 and 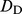 denote the events that a son or daughter, respectively, has the disease. We express the inter-lineage relative risk for sons as 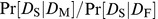 and that for daughters as 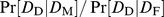.

Epidemiologic studies sometimes assemble very large case-control samples or cohorts at elevated risk [12], [13] and ascertain extensive family history data for affected families, enabling powerful comparisons of disease rates for maternal versus paternal lineages. Although studies related to birth defects have made use of multigenerational data [11] [14], [15], the huge consortia assembled for case-control and cohort studies of diseases like cancer [16] have thus far not probed for these less accessible genetic mechanisms.

The NIEHS Sister Study enrolled 50,884 women who each had a sister diagnosed with breast cancer. We here use data from that cohort to compare rates of breast cancer in maternal versus paternal grandmothers. We develop general results to relate the inter-lineage relative risks in progenitors to the inter-lineage relative risks in descendants. Under simplifying assumptions, we calculate how large a maternally-mediated prenatal effect or a parent-of-origin effect would have to be to explain any particular inter-lineage asymmetry and conclude that those causative relative risks would have to be substantial to produce the inter-lineage asymmetry evident in the Sister Study.

## Materials and Methods

We analyzed family history data for a large number of cases of breast cancer, to compare the risk of breast cancer in the maternal versus the paternal grandmother. The Sister Study [17] enrolled 50,884 women aged 35 to 74 in the United States and Puerto Rico between 2004 and 2009; each had a sister diagnosed with breast cancer. The unaffected sisters are being followed for newly incident breast cancer and other conditions. Participants completed a detailed family history questionnaire and were old enough that the data effectively encompass their grandmothers' lifetime risks. The Sister Study secured informed consent and was carried out with human-subjects approval and oversight from the NIEHS Institutional Review Board and the Copernicus Group Institutional Review Board.

To assess asymmetry, we calculated odds ratios (which approximate the relative risks for maternal versus paternal grandmothers) using families where exactly one of the two grandmothers had breast cancer, i.e., discordant grandmother pairs.

Using algebra we then derived formulae to assess the likely strength of the mechanisms that could underlie an observed asymmetry in family history. Foreseeing applications beyond breast cancer, we similarly derived expressions to assess the degree of asymmetry that those nonstandard mechanisms would produce in maternal versus paternal progenitors, and the degree of asymmetry produced in the offspring of affected male versus female individuals.

## Results

### The Sister Study

**Figure 1:**
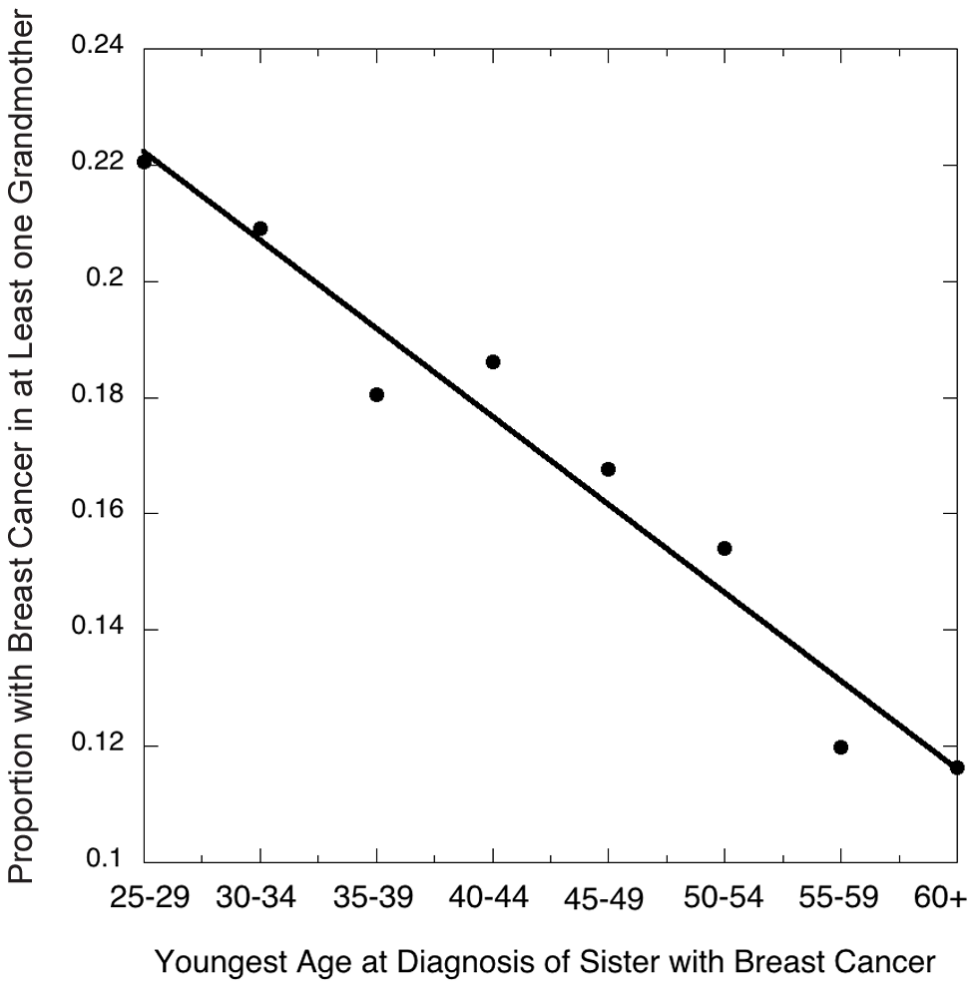
Risk of breast cancer in either grandmother as related to the youngest age at diagnosis of a granddaughter in the family studied (data taken from Table 1).

Our analysis used 32,929 women, each from a distinct family, where each woman was the full sister of a case and could report breast cancer history for both grandmothers. Of these grandmothers, 3046 on the maternal side (9%) and 2639 (8%) on the paternal side had developed breast cancer. These reported rates are in general agreement with expected rates for their birth cohort [18]. However, presumably reflecting heritability, the probability that at least one of the two grandmothers had developed breast cancer decreased as a function of the youngest age at diagnosis of a sister in the participating family (Figure 1, Table 2).

**Table 2:** Grandmothers' breast cancer history by age at breast cancer diagnosis of the youngest-onset grand-daughter.

Age at diagnosis of youngest-onset granddaughter	Which grandmothers had breast cancer				
	Neither	Only mother's mother	Only father's mother	Both	Inter-lineage grandmother odds ratio
<30	500	66	64	13	1.03
30–34	1313	177	167	20	1.06
35–39	3089	339	297	45	1.14
40–44	4925	569	476	78	1.20
45–49	6135	660	520	68	1.27
50–54	4940	484	373	49	1.30
55–59	3510	229	228	22	1.00
60–89	3151	203	195	24	1.04
TOTAL	27,563	2,727	2,320	319	1.18

**Figure 2:**
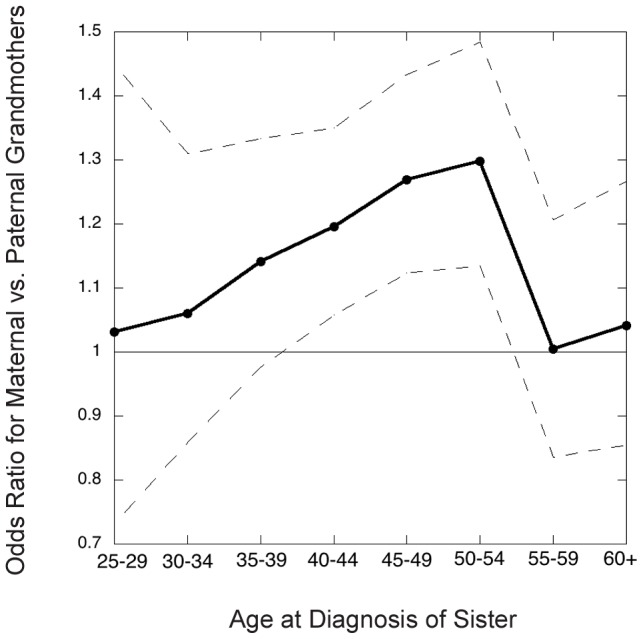
Grandmothers' odds ratio (maternal versus paternal) in the Sister Study as a function of youngest age at diagnosis of a granddaughter in the family studied. Dots connected by solid line segments are estimated odds ratios (approximately the relative risks); dashed lines connect 95% point-wise confidence limits.

Figure 2 shows the inter-lineage odds ratios (which approximate the relative risks, 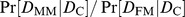) for maternal versus paternal grandmothers, using families where exactly one of the two grandmothers had breast cancer, i.e., discordant grandmother pairs. That is, we calculated the ratio of maternal to paternal grandmothers among the discordant pairs. The estimated overall odds ratio for a positive family history on the maternal versus paternal side was 1.18 (95% CI 1.11, 1.24, p<0.0001).

The inter-lineage odds ratio depended on the granddaughter's age at diagnosis (Figure 2), being most pronounced for cancers diagnosed in the age decade 45–54, i.e. in the perimenopausal years, and much less pronounced for later-diagnosed or very young onset cancers. We next wanted to relate the magnitude of the excess in maternal grandmothers to possible nonstandard genetic mechanisms.

### Quantifying Mechanisms That Can Underlie Asymmetry in Family History

#### An analytic result related to asymmetry in family histories

The degree of asymmetry in progenitors of affected individuals and the degree of asymmetry in offspring of affected individuals are closely related. Assume that any negative effect the disease has on reproductive success is equivalent in males and females and also that the relative risk for mothers versus fathers is the same as that for women versus men, and the risk for maternal grandmothers (grandfathers) is the same as that for paternal grandmothers (grandfathers). Straightforward manipulation of conditional probabilities proves the following result (Text S1).

Result 1: Under the above-stated assumptions, a) the inter-lineage relative risk for mothers with an affected offspring versus fathers with an affected offspring is the same as the inter-lineage relative risk for offspring with an affected mother versus those with an affected father, that is,
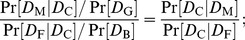



b), the inter-lineage relative risk for maternal grandmothers (grandfathers) with an affected grandchild versus paternal grandmothers (grandfathers) with an affected grandchild is the same as the inter-lineage relative risk for grandchildren with an affected maternal grandmother (grandfather) versus those with an affected paternal grandmother (grandfather). That is,
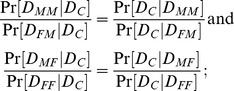



c) Similarly, provided the inter-lineage relative risk for sons equals that for daughters, the inter-lineage son (daughter) relative risk is the same as the inter-lineage parental relative risk for mothers versus fathers of affected offspring. That is,
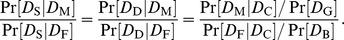



Note that Result 1 refers to a particular offspring or grandchild (for example, first-born or randomly selected) and thus the result does not apply to families selected because one out of some large number of progeny developed the disease.

#### Calculating genotype distributions across generations

To quantify inter-lineage relative risks, we adopt some additional simplifying assumptions: a rare outcome, random mating (relative to the di-allelic locus under study), Mendelian inheritance, Hardy-Weinberg equilibrium (HWE), and that the locus is not in linkage disequilibrium with and does not interact with another risk-related locus.

Let P be the 1×3 row vector containing an individual's autosomal genotype probabilities for minor allele counts 0, 1, 2 for a particular locus. Under Hardy-Weinberg Equilibrium (HWE), for a randomly sampled individual, 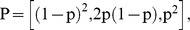 where 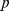 is the minor allele frequency. Now suppose sampling is instead based on the disease status of a designated relative, the proband case.

We need to introduce the concept of “risk relevant” genotypes. For maternally-mediated prenatal effects, those where the maternal genome affects the offspring's risk through prenatal influences on fetal development, the risk-relevant genotype at that locus is that of the affected proband's mother; and for autosomal genes with parent-of-origin effects the risk-relevant genotypes are those of both the proband and the proband's parents. Those risk-relevant genotypes will be distorted away from HWE for a locus related to risk. Other family members who are the progenitors or descendants of risk-relevant individuals are *not* risk-relevant to the proband, in that their genotypes at that locus are unrelated to the proband's risk conditional on the genotypes of risk-relevant individuals. However, their genotype distributions may still be distorted away from HWE, due to their relationship with the proband. The following result, proven in Text S2 (Table S1), facilitates calculation of autosomal genotype distributions for individuals who are not risk-relevant in this sense. We define the “index person” as the person whose parent's or whose offspring's genotype distribution is desired.

Result 2: Assume that the population meets the assumptions stated earlier. Let 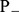 be the genotype distribution for an index person's parent (either mother or father),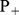 be the genotype distribution for an index person's offspring, and 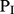 be the genotype distribution for the index person. Let 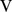 be the 3×3 matrix 
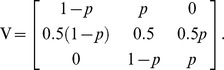



If 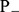 is not risk-relevant to the proband, then 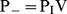. Similarly, if P_+_ is not risk-relevant to the proband, then 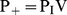.

The elements of the matrix V are the conditional probabilities for the offspring or parent to have a certain allele count, conditional on the index person having a certain allele count. Note that Result 2 holds under our assumptions even if the locus under study is not related to risk. This result allows the extrapolation of enrichment of the risk allele back to progenitors and forward to descendants.

### Maternal Effects

Suppose a genetic variant carried by the mother has a prenatal effect on fetal development, hence on later risk to her offspring. We quantify the asymmetry induced by such a maternal effect (Text S3).

**Figure 3:**
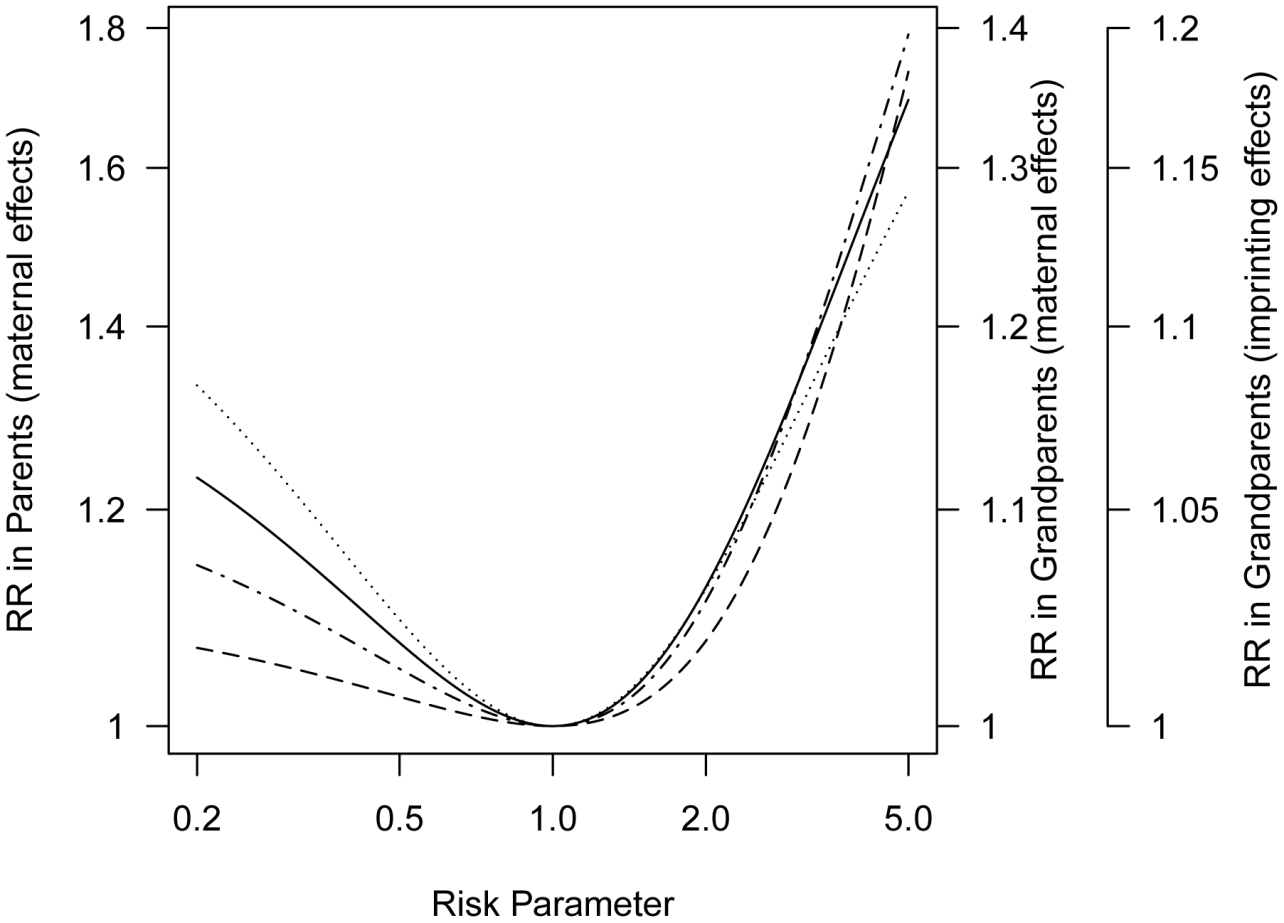
Progenitors relative risk (mothers versus fathers or maternal grandmothers versus paternal grandmothers) as a function of maternally mediated relative risk (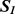) under a log-additive risk model (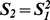), or the imprinting relative risk, *I*, for allele frequency 0.2 for a locus for which only a specific parental copy is expressed. The curve for parents for imprinting would overlay the curve for grandparents for a maternal effect.

Let the relative risk for the offspring of a mother with one (two) copies of the variant allele be 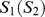 relative to the risk for the offspring of a mother with no copies. For simplicity we consider an outcome where gender itself does not influence risk and take 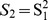 (log-additive risk model). Then mothers of affected offspring are enriched for the risk allele and so are their mothers, that is the maternal grandmothers. Consequently, the mothers themselves have greater risk than fathers whenever 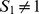 (Figure 3). Note that both causative and protective maternal effects produce increased risk in the maternal lineage, reflecting the fact that whichever of the two alleles confers higher risk will tend to be over-represented in the maternal lineage of a proband. Also, note that a given small inter-lineage relative risk is induced by a more extreme maternally-mediated relative risk 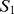.

This attenuation of 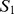 seen in the inter-lineage parent relative risk reflects the fact that the mother of a case could have inherited the susceptibility allele that influenced her offspring's risk from either her mother or her father and in the event it came from her father it would not have affected her own risk.

In general, we expect weaker asymmetry in the grandparental generation than in the parental generation. Assessment of risk to grandparents requires repeated use of the V matrix to calculate conditional genotype probabilities for *their* mothers, i.e., the sampled proband case's great-grandmothers (Text S3, Table S2). Because the relative risk for grandparents is a linear function of the relative risk for parents (Text S4), both sets of curves can be displayed in one figure with a simple scale change (Figure 3).

Returning to breast cancer, we see that if the modest perimenopausal asymmetry we saw (about 1.3) were due entirely to a maternally-acting SNP with a frequency of 10% and the effect obeyed a log-additive risk model, the relative risk for an offspring whose mother carries one copy of that SNP would be about 4.0.

Most diseases affect both males and females, so that, even if risk is maternally mediated, both grandmothers and grandfathers can contribute to the asymmetry analysis. In that event, the inter-lineage grandparental odds ratio (relative risk) is estimated by dividing the number of discordant pairs where the affected grandparent (either grandmother or grandfather) is on the maternal side by the number of discordant pairs where the affected grandparent is on the paternal side. If the condition is not rare so that some families contribute two discordant pairs, a within-cluster resampling approach [19] or generalized estimating equations approach [20] can accommodate family-based dependencies.

### Parent-of-Origin Effects

Another plausible source of asymmetry in family history involves parent-of-origin effects such as genetic imprinting. Such an effect was reported for breast cancer by Kong, et al [10] based on Icelandic family data. For simplicity we consider a situation where only the maternally-inherited copy at a risk-related locus is expressed (Table S2). Suppose the relative risk is *I* for offspring who carry a maternally-inherited copy of the variant compared to a risk of 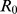 in individuals who do not (although for simplicity we assume 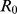 and *I* are the same for both sexes, they could be sex-specific) (Text S5, Table S3). For a parent-of-origin effect, here based on imprinting, maternal grandmothers of affected children show greater risk than paternal grandmothers whenever 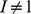 (Figure 3). Again the same set of curves can serve, as there is a simple scale change involved in moving from asymmetry induced by log-additive maternal effects to asymmetry induced by a parent-of-origin effect (Text S6). The observed grandparental relative risk of approximately 1.2 could reflect a polymorphic imprinted gene if the risk associated with a maternally-inherited allele (*I*) is 5.

### Inherited Mitochondrial Variants

Variants in mitochondrial DNA (mtDNA) can also produce asymmetry in families. A recent report found such a variant to be related to breast cancer risk in African-American women [9]. Since each person inherits virtually all their mitochondria from their mother, the asymmetry produced by this genetic mode of effect could show little or no diminution across generations of females, unless the mitochondria become heteroplasmic. The chain of mitochondrial inheritance is broken, however, by males: risk for the mother's father would on average return to the population risk because his mitochondria came from a separate maternal line. Returning to breast cancer, for a mitochondrial effect to explain the observed asymmetry it would have to confer about the same relative risk as that seen in the grandmothers, *i.e*., on the order of 1.2–1.3.

## Discussion

While case-control GWASs have revealed many SNPs related to susceptibility to complex diseases, nonstandard genetic mechanisms may also play a role. We considered three such mechanisms that can produce asymmetry in family histories: maternal genetic effects that influence risk via the prenatal environment; parent-of-origin effects, for example where the expression of an imprinted polymorphic gene variant depends on parental source; and effects of variants in the mitochondrial DNA, which are exclusively inherited from mothers. Our algebraic quantification of the relationship between inter-lineage asymmetry and its driving cause led us to conclude that, although the driving effect would be small if due to a mitochondrial variant, if a single variant acted through maternally-mediated prenatal effects or was subject to parent-of-origin effects, then that cause would be associated with a large relative risk, at least on the order of 4 or 5, for breast cancer.

Although a single-allele scenario seems unlikely, we wondered whether a single allele acting through a maternally-mediated genetic mechanism could explain the known increased risk seen in sisters. Under our simplifying homogeneity assumptions, one can show with a little added algebra that a single, log-additive maternal effect with a single-copy relative risk of 4.0 involving an allele with frequency about 0.07 would produce about the observed two-fold increased risk for sisters of cases.

Although we have focused our methods and analysis on progenitors and descendants, siblings, aunts and uncles would also be informative. A prenatal maternal effect and a parent-of-origin effect where only the maternal allele is expressed share an interesting feature: A half sibling of a case would have risk similar to that of a full sibling if the shared parent is the mother, but no increased risk if the shared parent is the father. A Danish study reported that pattern of asymmetry in half-brothers of cases with the birth defect cryptorchidism [21]. Under a maternally-mediated prenatal effect, because siblings share the same mother, the relative risk for the siblings of a mother with an affected child versus siblings of that child's father should be greater than 1. Thus, one could glean even more insight by comparing histories of maternal versus paternal blood-relative aunts and uncles, in addition to parents.

Several small studies have compared rates of breast cancer in maternal and paternal relatives. A registry-based Swedish study found no difference in maternal and paternal grandmothers but included fewer than a thousand breast cancer cases [22]. Two studies of healthy adults found an excess of cancer reported for female relatives [23], [24], but presumably more fathers than mothers were estranged and participants were not asked if they knew about particular relatives' disease histories. In the Sister Study, out of 44,307 families 1,843 reported about their paternal grandmother but not their maternal grandmother, while 4,895 reported about their maternal grandmother but not their paternal grandmother, suggesting that knowledge about maternal versus paternal grandmothers is differential. Unlike other studies, however, we restricted our analysis to families where the status of both was reported to minimize information bias.

Although our sample is large, with almost 33,000 families represented, cases reported among sisters and grandmothers were not generally validated clinically. Nevertheless, our participants are sisters of women with breast cancer; and they have proven themselves an informed and dedicated cohort, providing bio-samples, completing lengthy questionnaires and maintaining commitment to follow-up with a very low dropout rate. Moreover, though the study staff did not obtain medical records for cases whose sister enrolled in the Sister Study, we did request medical records for 1422 affected sisters who joined our family-based add-on “Two Sister Study” [25]. Their diagnosis of breast cancer was confirmed by medical records for all but 3 (who had lobular carcinoma *in situ*) of 1251.

We made some simplifying assumptions (HWE, Mendelian transmission, random mating, rare disease, effect of only a single locus) and thus our figures depict idealized settings, which are not fully appropriate for the Sister Study. There are also effects secondary to ascertainment. The Sister Study is more likely to enroll unaffected women from larger families who have lower genetic risks. Consider two families, each with a single daughter with breast cancer and suppose one family has 10 daughters and the second has only two daughters. The first family is more likely to be in our study because any one of the 9 unaffected daughters can join, but the same large family has demonstrated lower genetic risk with only one of 10 affected, compared to the second family with 1 of 2 affected. The Sister Study consequently would have sampled families with less genetic enrichment for the allele under study than the two-fold increase presumed. Because this ascertainment effect should distort the inter-lineage asymmetry toward the null, the estimate of 4 for a maternally-mediated prenatal effect may be too low.

We implicitly assumed that the reported father is the biological father, but reported paternity can be incorrect. However, out of a subset of 602 families in the Two Sister Study where DNA was acquired, only 5 fathers failed the paternity test. Moreover, the observed strong pattern where asymmetry was related to the age at diagnosis of the granddaughter could not be explained by misidentified paternity. Another issue is that in a large series of cases such as the Sister Study, some affected sisters may unknowingly have been adopted; however, we expect that proportion to be small. Also, unaware adoptees would report the wrong history on both the maternal and the paternal side, driving estimates toward symmetry.

Reporting bias and self-selection may be at work. One might expect a woman with both a sister and a mother with breast cancer to be more likely to join the Sister Study than a woman with only an affected sister. Also, women whose mother had breast cancer may undergo more regular screening. However, the rate of breast cancer reported for mothers was about 18%, which does not exceed expectation based on having a first degree relative with breast cancer.

With combined data including as many as 45,000 cases, large consortial efforts are underway [16] to study the genetics of cancers and other complex diseases. We believe that important clues could be elicited by also studying asymmetries in the reported family histories for those cases.

Although we saw evidence for inter-lineage asymmetry based on the family histories from the Sister Study participants, with more breast cancer in the maternal lineage, one cannot differentiate among the three nonstandard mechanisms using only phenotypic family histories of affected individuals. Only family-based genotype data will enable an investigator to identify the genetic mechanism and identify relevant variants [26], [27].

In summary, susceptibility to complex disease can be influenced by inherited autosomal gene variants, as most GWASs assume, but can also be influenced by sex-linked genes, maternally-mediated prenatal effects, parent-of-origin effects, and mitochondrial variants. These under-studied genetic mechanisms are best explored through family studies. Yet even without access to genetic data on family members, evidence that those phenomena play a role can be adduced through careful analyses of family history data from large assemblages of cases. Breast cancer appears to be subject to genetic mechanisms that produce family history asymmetry, particularly when diagnosed in the perimenopausal years.

## Supporting Information

Table S1 Probability of each case-parents triad genotype in a population with random mating, Mendelian inheritance and Hardy-Weinberg equilibrium at the locus under study.
(DOCX)

Table S2Probability of each case-parents triad genotype selected from a population with random mating, Mendelian inheritance and Hardy-Weinberg equilibrium at the locus under study conditional on the presence of an affected child when risk to the child depends only on the maternal genotype with risk vector 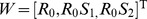 (symbols defined in the main text).(DOCX)

Table S3Probability of each case-parents triad genotype selected from a population with random mating, Mendelian inheritance and Hardy-Weinberg equilibrium at the locus under study conditional on the presence of an affected child when risk to a child depends only having inherited a copy of the variant allele from its mother with risk vector 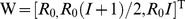 (symbols defined in the main text).(DOCX)

Text S1 Relationship between asymmetry in progenitors and progeny.
(DOCX)

Text S2 Carrying genotype distributions across generations.
(DOCX)

Text S3 Asymmetry in parents and grandparents due to maternal effect.
(DOCX)

Text S4 Relationship between parent and grandparent asymmetry induced by a maternal effect.
(DOCX)

Text S5 Asymmetry produced by an imprinted genetic variant.
(DOCX)

Text S6 Relationship between parental asymmetry induced by maternal effects and grandparental asymmetry induced by a parent-of-origin effect.
(DOCX)
