# Supplementary material for: Asymmetry in Family History Implicates Nonstandard Genetic Mechanisms: Application to the Genetics of Breast Cancer
Source: PLoS Genet. 2014 Mar 20;10(3):e1004174. doi: 10.1371/journal.pgen.1004174 (PMC3961172; doi:10.1371/journal.pgen.1004174)
Supplement: Table S1 — Probability of each case-parents triad genotype in a population with random mating, Mendelian inheritance and Hardy-Weinberg equilibrium at the locus under study. (DOCX) [file pgen.1004174.s001.docx]

**Table S1**. Probability of each case-parents triad genotype in a population with random mating, Mendelian inheritance and Hardy-Weinberg equilibrium at the locus under study.

| Genotypes (number of variant alleles) | | | Cell probabilities: |
| --- | --- | --- | --- |
| Mother (*M*) | Father (*F*) | Child (C) | $\Pr\left[ M,F,C \right]=P r\left[ M \right] P r\left[ F \right] P r\left[ C\vert M,F \right]$ |
|  |  |  |  |
| 2 | 2 | 2 | $p^{4}$ |
|  |  |  |  |
| 2 | 1 | 2 | $p^{3}q$ |
| 2 | 1 | 1 | $p^{3}q$ |
| 1 | 2 | 2 | $p^{3}q$ |
| 1 | 2 | 1 | $p^{3}q$ |
|  |  |  |  |
| 2 | 0 | 1 | $p^{2}q^{2}$ |
| 0 | 2 | 1 | $p^{2}q^{2}$ |
|  |  |  |  |
| 1 | 1 | 2 | $p^{2}q^{2}$ |
| 1 | 1 | 1 | $2p^{2}q^{2}$ |
| 1 | 1 | 0 | $p^{2}q^{2}$ |
|  |  |  |  |
| 1 | 0 | 1 | $pq^{3}$ |
| 1 | 0 | 0 | $pq^{3}$ |
| 0 | 1 | 1 | $pq^{3}$ |
| 0 | 1 | 0 | $pq^{3}$ |
|  |  |  |  |
| 0 | 0 | 0 | $q^{4}$ |
|  |  |  |  |

Notes:

1. $p$ is the frequency of the variant allele; $q\equiv1-p$ .

$2$. The sum of the cell probabilities is one since $p^{4}+4p^{3}q+6p^{2}q^{2}+4pq^{3}+q^{4}=\left( p+q \right)^{4}=1$

3. $\Pr\left[ C=c \right]$ is calculated by summing all cell probabilities where $C=c$ for $c\in\{0,1,2\}$. The resulting row vector giving the genotype distribution among children is $P_{C}=\left[ q^{2}, 2pq, p^{2} \right]$. Analogous calculations give $\Pr\left[ M=m \right]$ and $\Pr\left[ F=f \right]$ and show that the genotype distribution among mothers and that among fathers are the same as the genotype distribution among children; in other words, $P_{M}=P_{F}=P_{C}=\left[ q^{2}, 2pq, p^{2} \right]$, the Hardy-Weinberg equilibrium distribution.

4. The 3×3 matrix $V$ containing $\Pr[M=m|C=c]$ as the entry in row *c* and column *m*, as given in the main text, is calculated by first calculating $\Pr[M=m,C=c]$ by summing all cell probabilities where $M=m$ and $C=c$ and then dividing those probabilities by $\Pr[C=c]$. For example, $\Pr\left[ M=1 | C=1 \right]= \frac{\Pr[M=1,C=1]}{Pr[C=1]}=\frac{\Pr\left[ M=1,F=0, C=1 \right]+\Pr\left[ M=1,F=1, C=1 \right]+\Pr[M=1,F=2, C=1]}{Pr[C=1]}=\frac{pq^{3}+2p^{2}q^{2}+p^{3}q}{2pq}=\frac{pq(p^{2}{+2pq+q}^{2})}{2pq}=0.5$
