# Supplementary material for: Asymmetry in Family History Implicates Nonstandard Genetic Mechanisms: Application to the Genetics of Breast Cancer
Source: PLoS Genet. 2014 Mar 20;10(3):e1004174. doi: 10.1371/journal.pgen.1004174 (PMC3961172; doi:10.1371/journal.pgen.1004174)
Supplement: Table S2 — Probability of each case-parents triad genotype selected from a population with random mating, Mendelian inheritance and Hardy-Weinberg equilibrium at the locus under study conditional on the presence of an affected child when risk to the child depends only on the maternal genotype with risk vector (symbols defined in the main text). (DOCX) [file pgen.1004174.s002.docx]

**Table S2**. Probability of each case-parents triad genotype selected from a population with random mating, Mendelian inheritance and Hardy-Weinberg equilibrium at the locus under study conditional on the presence of an affected child when risk to the child depends only on the maternal genotype with risk vector $W=\left[ R_{0}, R_{0}S_{1},R_{0}S_{2} \right]^{T}$ (symbols defined in the main text).

| Genotypes (number of variant alleles) | | | Cell probabilities: |
| --- | --- | --- | --- |
| Mother (*M*) | Father (*F*) | Child (C) | $\Pr\left[ MFC\vert D_{C} \right]=\left( P r\left[ MFC \right] P r\left[ D_{C}\vert MFC \right] \right)/{P r\left[ D_{C} \right]}$ |
|  |  |  |  |
| 2 | 2 | 2 | ${S_{2}p}^{4}K^{-1}$ |
|  |  |  |  |
| 2 | 1 | 2 | ${S_{2}p}^{3}qK^{-1}$ |
| 2 | 1 | 1 | ${S_{2}p}^{3}qK^{-1}$ |
| 1 | 2 | 2 | ${S_{1}p}^{3}qK^{-1}$ |
| 1 | 2 | 1 | ${S_{1}p}^{3}qK^{-1}$ |
|  |  |  |  |
| 2 | 0 | 1 | ${S_{2}p}^{2}q^{2}K^{-1}$ |
| 0 | 2 | 1 | $p^{2}q^{2}K^{-1}$ |
|  |  |  |  |
| 1 | 1 | 2 | ${S_{1}p}^{2}q^{2}K^{-1}$ |
| 1 | 1 | 1 | $2S_{1}p^{2}q^{2}K^{-1}$ |
| 1 | 1 | 0 | $S_{1}p^{2}q^{2}K^{-1}$ |
|  |  |  |  |
| 1 | 0 | 1 | $S_{1}pq^{3}K^{-1}$ |
| 1 | 0 | 0 | $S_{1}pq^{3}K^{-1}$ |
| 0 | 1 | 1 | $pq^{3}K^{-1}$ |
| 0 | 1 | 0 | $pq^{3}K^{-1}$ |
|  |  |  |  |
| 0 | 0 | 0 | $q^{4}K^{-1}$ |
|  |  |  |  |

Notes:

1. $K^{-1}$ is the constant that makes the conditional cell probabilities sum to one; $K={S_{2}p}^{2}+2S_{1}pq+q^{2}$. The baseline risk $R_{0}$ cancels out of expressions for the conditional cell probabilities.

2. $\Pr\left[ M=m|D_{C} \right]$ is calculated by summing all cell probabilities where $M=m$ for $m\in\{0,1,2\}$. The resulting row vector giving the genotype distribution among mothers of affected children is $P_{M|D_{C}}=\left[ q^{2}K^{-1}, 2S_{1}pqK^{-1}, {S_{2}p}^{2}K^{-1} \right]$.

3. $\Pr\left[ F=f|D_{C} \right]$ is calculated by summing all cell probabilities where $F=f$ for $f\in\{0,1,2\}$. The resulting row vector giving the genotype distribution among fathers of affected children is $P_{F|D_{C}}=\left[ q^{2}, 2pq, p^{2} \right]$, the Hardy-Weinberg equilibrium distribution.
