# Supplementary material for: Asymmetry in Family History Implicates Nonstandard Genetic Mechanisms: Application to the Genetics of Breast Cancer
Source: PLoS Genet. 2014 Mar 20;10(3):e1004174. doi: 10.1371/journal.pgen.1004174 (PMC3961172; doi:10.1371/journal.pgen.1004174)
Supplement: Table S3 — Probability of each case-parents triad genotype selected from a population with random mating, Mendelian inheritance and Hardy-Weinberg equilibrium at the locus under study conditional on the presence of an affected child when risk to a child depends only having inherited a copy of the variant allele from its mother with risk vector (symbols defined in the main text). (DOCX) [file pgen.1004174.s003.docx]

**Table S3**. Probability of each case-parents triad genotype selected from a population with random mating, Mendelian inheritance and Hardy-Weinberg equilibrium at the locus under study conditional on the presence of an affected child when risk to a child depends only having inherited a copy of the variant allele from its mother with risk vector $W=\left[ R_{0}, R_{0}(I+1)/2,R_{0}I \right]^{T}$ (symbols defined in the main text).

| Genotypes (number of variant alleles) | | | Cell probabilities: |
| --- | --- | --- | --- |
| Mother (*M*) | Father (*F*) | Child (C) | $\Pr\left[ MFC\vert D_{C} \right]=\left( P r\left[ MFC \right] P r\left[ D_{C}\vert MFC \right] \right)/{P r\left[ D_{C} \right]}$ |
|  |  |  |  |
| 2 | 2 | 2 | ${Ip}^{4}Q^{-1}$ |
|  |  |  |  |
| 2 | 1 | 2 | ${Ip}^{3}qQ^{-1}$ |
| 2 | 1 | 1 | ${Ip}^{3}qQ^{-1}$ |
| 1 | 2 | 2 | ${Ip}^{3}qQ^{-1}$ |
| 1 | 2 | 1 | $p^{3}qQ^{-1}$ |
|  |  |  |  |
| 2 | 0 | 1 | ${Ip}^{2}q^{2}Q^{-1}$ |
| 0 | 2 | 1 | $p^{2}q^{2}Q^{-1}$ |
|  |  |  |  |
| 1 | 1 | 2 | ${Ip}^{2}q^{2}Q^{-1}$ |
| 1 | 1 | 1 | $(I+1)p^{2}q^{2}Q^{-1}$ |
| 1 | 1 | 0 | $p^{2}q^{2}Q^{-1}$ |
|  |  |  |  |
| 1 | 0 | 1 | $Ipq^{3}Q^{-1}$ |
| 1 | 0 | 0 | $pq^{3}Q^{-1}$ |
| 0 | 1 | 1 | $pq^{3}Q^{-1}$ |
| 0 | 1 | 0 | $pq^{3}Q^{-1}$ |
|  |  |  |  |
| 0 | 0 | 0 | $q^{4}Q^{-1}$ |
|  |  |  |  |

Notes:

1. $Q^{-1}$ is the constant that makes the conditional cell probabilities sum to one; $Q=Ip+q$. The baseline risk $R_{0}$ cancels out of expressions for the conditional cell probabilities.

2. $\Pr\left[ M=m|D_{C} \right]$ is calculated by summing all cell probabilities where $M=m$ for $m\in\{0,1,2\}$. The resulting row vector giving the genotype distribution among mothers of affected children is $P_{M|D_{C}}=\left[ q^{2}Q^{-1}, (I+1)pqQ^{-1}, {Ip}^{2}Q^{-1} \right]$. An analogous calculation shows that the genotype distribution among affected children is exactly the same as that among their mothers, that is, $P_{M|D_{C}}=P_{C|D_{C}}$.

3. $\Pr\left[ F=f|D_{C} \right]$ is calculated by summing all cell probabilities where $F=f$ for $f\in\{0,1,2\}$. The resulting row vector giving the genotype distribution among fathers of affected children is $P_{F|D_{C}}=\left[ q^{2}, 2pq, p^{2} \right]$, the Hardy-Weinberg equilibrium distribution.
