# Supplementary material for: Asymmetry in Family History Implicates Nonstandard Genetic Mechanisms: Application to the Genetics of Breast Cancer
Source: PLoS Genet. 2014 Mar 20;10(3):e1004174. doi: 10.1371/journal.pgen.1004174 (PMC3961172; doi:10.1371/journal.pgen.1004174)
Supplement: Text S1 — Relationship between asymmetry in progenitors and progeny. (DOCX) [file pgen.1004174.s004.docx]

**Text S1: Relationship between asymmetry in progenitors and progeny**

Result 1: Under the above-stated assumption, a) provided the relative risk for mothers versus fathers is the same as that for females versus males, the inter-lineage relative risk for mothers with a particular offspring affected versus fathers with a particular offspring affected is the same as the inter-lineage relative risk for offspring with an affected mother versus those with an affected father, that is,

$$\frac{{\Pr\left[ D_{M} | D_{C} \right]}/{\Pr\left[ D_{G} \right]}}{{\Pr\left[ D_{F} | D_{C} \right]}/{\Pr\left[ D_{B} \right]}}=\frac{\Pr\left[ D_{C} | D_{M} \right]}{\Pr\left[ D_{C} | D_{F} \right]};$$

b) provided the risk for maternal grandmothers (grandfathers) is the same as that for paternal grandmothers (grandfathers), the inter-lineage relative risk for maternal grandmothers (grandfathers) with a particular grandchild affected versus paternal grandmothers (grandfathers) with a particular grandchild affected is the same as the inter-lineage relative risk for grandchildren with an affected maternal grandmother (grandfather) versus those with an affected paternal grandmother (grandfather) . That is,

$$\frac{\Pr\left[ D_{MM}|D_{C} \right]}{\Pr\left[ D_{FM}|D_{C} \right]}=\frac{\Pr\left[ D_{C}|D_{MM} \right]}{\Pr\left[ D_{C}|D_{FM} \right]} \mathrm{and} \frac{\Pr\left[ D_{MF}|D_{C} \right]}{\Pr\left[ D_{FF}|D_{C} \right]}=\frac{\Pr\left[ D_{C}|D_{MF} \right]}{\Pr\left[ D_{C}|D_{FF} \right]};$$

c) Similarly, provided the inter-lineage relative risk for sons equals that for daughters, the inter-lineage son (daughter) relative risk is the same as the inter-lineage parental relative risk for mothers versus fathers of affected offspring. That is,

$$\frac{\Pr\left[ D_{S} | D_{M} \right]}{\Pr\left[ D_{S} | D_{F} \right]}=\frac{\Pr\left[ D_{D} | D_{M} \right]}{\Pr\left[ D_{D} | D_{F} \right]}=\frac{{\Pr\left[ D_{M} | D_{C} \right]}/{\Pr\left[ D_{G} \right]}}{{\Pr\left[ D_{F} | D_{C} \right]}/{\Pr\left[ D_{B} \right]}}.$$

First, we consider the equality of the inter-lineage parent and the inter-lineage offspring relative risks for parents/offspring of an affected individual. Let $D_{C}$, $D_{M}$, $D_{F}$ denote the events that the offspring, mother, father has the disease, respectively, with analogous notation for other relatives. Let $D_{G}$, $D_{B}$ denote the events that a female (girl), male (boy) in the population has the disease, respectively. Using this notation, the inter-lineage parent relative risk as defined in the manuscript is:

$\frac{{\Pr\left[ D_{M} | D_{C} \right]}/{\Pr\left[ D_{G} \right]}}{{\Pr\left[ D_{F} | D_{C} \right]}/{\Pr\left[ D_{B} \right]}}.$ (A1)

Now, using standard probability calculations,

$\Pr\left[ D_{M} | D_{C} \right]=\frac{\Pr\left[ D_{C} | D_{M} \right]\Pr\left[ D_{M} \right]}{\Pr\left[ D_{C} \right]}$, (A2)

and similarly for $\Pr\left[ D_{F} | D_{C} \right]$. In addition, we assume that the relative risk in women versus men in the population is the same as that in mothers versus fathers, *i.e.*, ${\Pr\left[ D_{G} \right]}/{\Pr\left[ D_{B} \right]}={\Pr\left[ D_{F} \right]}/{\Pr\left[ D_{M} \right]}$. Substituting all these relationships into A1 and simplifying shows that

$\frac{{\Pr\left[ D_{M} | D_{C} \right]}/{\Pr\left[ D_{G} \right]}}{{\Pr\left[ D_{F} | D_{C} \right]}/{\Pr\left[ D_{B} \right]}}=\frac{\Pr\left[ D_{C} | D_{M} \right]}{\Pr\left[ D_{C} | D_{F} \right]}.$ (A3)

The right-hand side represents the inter-lineage offspring relative risk. Thus, the inter-lineage parent relative risks and the inter-lineage offspring relative risks are the same.

An analogous results hold for grandparents and grandchildren. The inter-lineage grandmother relative risk as defined in the manuscript is:

$\frac{\Pr\left[ D_{\mathrm{MM}} | D_{C} \right]}{\Pr\left[ D_{\mathrm{FM}} | D_{C} \right]}.$ (A4)

Substituting the analogues of A2 for $\Pr\left[ D_{\mathrm{MM}} | D_{C} \right]$ and for $\Pr\left[ D_{\mathrm{FM}} | D_{C} \right]$ into the right-hand side of A3, using the assumption that $\Pr\left[ D_{\mathrm{MM}} \right]=\Pr\left[ D_{\mathrm{FM}} \right]$, and simplifying yields:

$$\frac{\Pr\left[ D_{MM}|D_{C} \right]}{\Pr\left[ D_{FM}|D_{C} \right]}=\frac{\Pr\left[ D_{C}|D_{MM} \right]}{\Pr\left[ D_{C}|D_{FM} \right]}.$$

The same argument applied to grandfathers shows:

$$\frac{\Pr\left[ D_{MF}|D_{C} \right]}{\Pr\left[ D_{FF}|D_{C} \right]}=\frac{\Pr\left[ D_{C}|D_{MF} \right]}{\Pr\left[ D_{C}|D_{FF} \right]}.$$

These results demonstrate that the inter-lineage grandparent relative risks are the same as the inter-lineage grandchildren relative risks. Note that we have not needed to make any assumptions except stability of the relative risks over time. The same results hold for relative odds instead of relative risks.

Next, we consider the equality of the inter-lineage parent relative risk in A3 and the inter-lineage relative risk for the sons of affected mothers versus sons of affected fathers. Writing $D_{S}$, $D_{D}$ for the events that the son, daughter has the disease, respectively, the inter-lineage relative risk for sons as defined in the manuscript is:

$\frac{\Pr\left[ D_{S} | D_{M} \right]}{\Pr\left[ D_{S} | D_{F} \right]}.$ (A4)

We assume that the inter-lineage relative risk for sons is the same as that for daughters, that is,

$\frac{\Pr\left[ D_{S} | D_{M} \right]}{\Pr\left[ D_{S} | D_{F} \right]}=\frac{\Pr\left[ D_{D} | D_{M} \right]}{\Pr\left[ D_{D} | D_{F} \right]}$*.* (A5)

Equality A5 can be algebraically re-arranged to show that $\frac{\Pr\left[ D_{D} | D_{M} \right]}{\Pr\left[ D_{S} | D_{M} \right]}=\frac{\Pr\left[ D_{D} | D_{F} \right]}{\Pr\left[ D_{S} | D_{F} \right]}$. Let $R$ denote the common value of these ratios. Alternate re-arrangements of A5 show that $\Pr\left[ D_{D} | D_{M} \right]=\frac{\Pr\left[ D_{D} | D_{F} \right]}{\Pr\left[ D_{S} | D_{F} \right]}\Pr\left[ D_{S} | D_{M} \right]=R\Pr\left[ D_{S} | D_{M} \right]$ and similarly $\Pr\left[ D_{D} | D_{F} \right]=R\Pr\left[ D_{S} | D_{F} \right]$. Because an affected child must be either a son or a daughter, we also have $\Pr\left[ D_{C} | D_{M} \right]=\Pr\left[ D_{S} | D_{M} \right]+\Pr\left[ D_{D} | D_{M} \right]$ and a similar relationship for $\Pr\left[ D_{C} | D_{F} \right]$.

Consider the inter-lineage offspring relative risk, namely, $\frac{\Pr\left[ D_{C} | D_{M} \right]}{\Pr\left[ D_{C} | D_{F} \right]}$. From A3 we know that it is equal to the inter-lineage parental relative risk. What remains to be shown is that the inter-lineage offspring relative risk is equal to the inter-lineage relative risk for sons, or, in symbols, that $\frac{\Pr\left[ D_{C} | D_{M} \right]}{\Pr\left[ D_{C} | D_{F} \right]}=\frac{\Pr\left[ D_{S} | D_{M} \right]}{\Pr\left[ D_{S} | D_{F} \right]}$. Using the various relationships stated in the previous paragraph, we can write:

$$\frac{\Pr\left[ D_{C} | D_{M} \right]}{\Pr\left[ D_{C} | D_{F} \right]}=\frac{\Pr\left[ D_{S} | D_{M} \right]+\Pr\left[ D_{D} | D_{M} \right]}{\Pr\left[ D_{S} | D_{F} \right]+\Pr\left[ D_{D} | D_{F} \right]}=\frac{\Pr\left[ D_{S} | D_{M} \right]+R\Pr\left[ D_{S} | D_{M} \right]}{\Pr\left[ D_{S} | D_{F} \right]+R\Pr\left[ D_{S} | D_{F} \right]}=\frac{\Pr\left[ D_{S} | D_{M} \right]}{\Pr\left[ D_{S} | D_{F} \right]}.$$

Thus, assuming that A5 holds, the inter-lineage offspring relative risk is equal to the inter-lineage relative risk for sons. Under A5, the relationship must also hold for the inter-lineage relative risk for daughters.
