# Supplementary material for: Asymmetry in Family History Implicates Nonstandard Genetic Mechanisms: Application to the Genetics of Breast Cancer
Source: PLoS Genet. 2014 Mar 20;10(3):e1004174. doi: 10.1371/journal.pgen.1004174 (PMC3961172; doi:10.1371/journal.pgen.1004174)
Supplement: Text S2 — Carrying genotype distributions across generations. (DOCX) [file pgen.1004174.s005.docx]

**Text S2.** **Carrying genotype distributions across generations.**

**Restating the result:**

**Result 2**: Assume that the population meets the assumptions stated earlier. Let $P_{-}$ be the genotype distribution for an index person’s parent (either mother or father), $P_{+}$ be the genotype distribution for an index person’s offspring, and $P_{I}$ be the genotype distribution for the index person. Let $V$ be the 3×3 matrix

$$V=\left[ \begin{matrix} 1-p & p & 0 \\ 0.5(1-p) & 0.5 & 0.5p \\ 0 & 1-p & p \end{matrix} \right].$$

If $P_{-}$is not risk-relevant to the proband, then $P_{-}=P_{I}V$. Similarly, if $P_{+}$ is not risk-relevant to the proband, then $P_{+}=P_{I}V$.

For ease of presentation when demonstrating Result 2, we focus on relationships between mother and child as the consecutive generations; however, because this parent-offspring pair represents any consecutive generations, the proof applies with full generality. First, we consider the population as a whole to show the relationships hold for unselected individuals and to derive the $V$ matrix. Subsequently, we consider selecting families based on probands with disease in order to calculate genotype distributions for parents or offspring who are not risk-relevant to the proband. Let *C, M,* and *F* denote the number of copies of the minor allele carried by the offspring, the mother, and the father, respectively. Again we define the “index person” as the person whose parent’s or whose offspring’s genotype distribution is desired.

Consider the situation where the index person (either mother or child) is sampled at random from the entire population. Assume first that the child is the index person and that we know $P_{C}$, the 1×3 row vector containing $\Pr\left[ C=c \right]$ for $c=0,1,2$. To calculate $P_{M}$, the 1×3 row vector containing $\Pr\left[ M=m \right]$ for $m=0,1,2$, we use the following probability relationship:

$\Pr\left[ M=m \right]=\sum_{c} \left( \Pr\left[ C=c \right]Pr[M=m|C=c] \right)$ for $m=0,1,2$.

Expressing this relationship compactly using matrix notation, we have $P_{M}=P_{C}U_{-}$, where $U_{-}$ is a 3×3 matrix containing $Pr[M=m|C=c]$ as the entry in row *c* and column *m*. Assume next that the mother is the index person and that we know $P_{M}$. To calculate $P_{C}$, we use

$\Pr\left[ C=c \right]=\sum_{m} \left( \Pr\left[ M=m \right]Pr[C=c|M=m] \right)$ for $c=0,1,2$,

or, equivalently, $P_{C}=P_{M}U_{+}$, where $U_{+}$ is a 3×3 matrix containing $Pr[C=c|M=m]$ as the entry in row *m* and column *c*.

Now, under our assumptions, $U_{-}=U_{+}=V$ where $V\equiv\left[ \begin{matrix} 1-p & p & 0 \\ 0.5(1-p) & 0.5 & 0.5p \\ 0 & 1-p & p \end{matrix} \right]$. To see the equality of these matrices, one calculates (we adopt an abbreviated notation here) $U_{-}=Pr[M|C]$ and $U_{+}=\Pr\left[ C | M \right]$directly from the joint distribution $Pr[M,F,C]$ (Table S1) via $\Pr\left[ C | M \right]=\frac{\sum_{f} Pr[M,F,C]}{\sum_{f} \sum_{c} Pr[M,F,C]}$ and $\Pr\left[ M | C \right]=\frac{\sum_{f} Pr[M,F,C]}{\sum_{f} \sum_{m} Pr[M,F,C]}$.

Thus, for index persons selected at random from the population, when an offspring is the index person, we can calculate the genotype distribution of the mother as $P_{M}=P_{C}V$, which becomes $P_{-}=P_{I}V$ when re-expressed in the general notation for adjacent generations. Similarly, when a mother is the index person, $P_{C}=P_{M}V$, or equivalently, $P_{+}=P_{I}V$. Reassuringly, under our assumptions and provided that the index person is randomly selected from the population, the matrix $V$ translates a HWE genotype distribution in the index person to the same HWE distribution in a parent (father or mother) or an offspring.

Consider now the situation where a mother/offspring pair comes from a family selected because a particular proband family member is affected, designated event $D$. Suppose we want to extrapolate from the offspring’s genotype distribution $P_{C|D}$to that of their mother, taking the offspring as the index person. $P_{C|D}$is the 1×3 row vector containing $\Pr\left[ C=c|D \right]$ for $c=0,1,2$. We calculate $P_{M|D}$, the 1×3 row vector containing $\Pr\left[ M=m|D \right]$ for $m=0,1,2$, using

$\Pr\left[ M=m|D \right]=\sum_{c} \left( \Pr\left[ C=c|D \right]Pr[M=m|C=c,D] \right)$,

or, $P_{M|D}=P_{C|D}B_{-}$ where $B_{-}$ is a 3×3 matrix containing $Pr[M=m|C=c,D]$ as the entry in row *c* and column *m*. Now, $B_{-}=V$ whenever $Pr[M=m|C=c,D]$ =$Pr[M=m|C=c]$ for all *m* and *c*. Using well-known probability relationships, one can write

$\Pr\left[ M | C,D \right]=\frac{\Pr\left[ D | M,C \right]Pr[M|C]Pr[C]}{\Pr\left[ D | C \right]Pr[C]}=\frac{\Pr\left[ D | M,C \right]}{\Pr\left[ D | C \right]}Pr[M|C]$.

Thus, $B_{-}=V$ if and only if $\Pr\left[ D | M,C \right]=\Pr\left[ D | C \right]$, that is, if and only if the mother’s genotype is not risk-relevant to the proband, as defined in the text. Thus, if the genotype of the parent of the index person is not risk-relevant to the proband, then $P_{-}=P_{I}V$. A similar argument taking the mother as the index person shows that, if the genotype of the offspring of the index person is not risk-relevant to the proband, then $P_{+}=P_{I}V$. This completes the proof of Result 2.
