# Supplementary material for: Asymmetry in Family History Implicates Nonstandard Genetic Mechanisms: Application to the Genetics of Breast Cancer
Source: PLoS Genet. 2014 Mar 20;10(3):e1004174. doi: 10.1371/journal.pgen.1004174 (PMC3961172; doi:10.1371/journal.pgen.1004174)
Supplement: Text S3 — Asymmetry in parents and grandparents due to maternal effect. (DOCX) [file pgen.1004174.s006.docx]

**Text S3. Asymmetry in parents and grandparents due to maternal effects.**

For a given underlying risk model, calculating the inter-lineage parent or grandparent relative risks involves three steps: (1) calculating the genotype distribution in the relevant risk-determining generation in each lineage conditional on the offspring disease status; (2) calculating the lineage-specific risks under the assumed risk model; (3) calculating the inter-lineage relative risk based on the lineage-specific risks. If assumptions are met, use of the matrix $V$ simplifies calculations in step 1. Step 2 is accomplished by taking the product of the 1×3 row vector containing the genotype distribution from step 1 and the 3×1 column vector containing genotype-specific risks of disease under the assumed risk model.

**Parents.**

From S1, the inter-lineage parent relative risk is:

$\mathbb{P=}\frac{{\Pr\left[ D_{M} | D_{C} \right]}/{\Pr\left[ D_{G} \right]}}{{\Pr\left[ D_{F} | D_{C} \right]}/{\Pr\left[ D_{B} \right]}}.$ (C1)

Thus, we need to calculate risk to mothers and to fathers of affected children as well as to males and females in the population. We assume the population meets the assumptions stated in the manuscript. Under maternal effects, mothers’ genotypes alone determine the risk to their children so that grandmothers’ genotypes are not risk-relevant. Thus, $\Pr\left[ D_{M} | {MM=m,D}_{C} \right]=\Pr\left[ D_{M} | \mathrm{MM}=m \right]$ for $m=0,1,2$ so that $\Pr\left[ D_{M} | D_{C} \right]=\sum_{m} \Pr\left[ MM=m | D_{C} \right]\Pr\left[ D_{M} | \mathrm{MM}=m \right]=P_{MM|D_{c}}W_{G}$. Here $P_{MM|D_{c}}$denotes a 1×3 genotype probability vector containing $\Pr\left[ MM=m | D_{C} \right]$ for $m=0,1,2$ and $W_{G}$ (G for girl) denotes a 3×1 risk vector containing $\Pr\left[ D_{M} | \mathrm{MM}=m \right]$ for $m=0,1,2$. (Of course, under maternal effects, $\Pr\left[ D_{M} | \mathrm{MM}=m \right]$ is the risk for a female offspring given her mother’s genotype.) Analogous considerations yield: $\Pr\left[ D_{F} | D_{C} \right]=P_{FM|D_{c}}W_{B}$ where $W_{B}$ (B for boy) denotes a risk vector containing $\Pr\left[ D_{F} | \mathrm{FM}=m \right]$, the risk for a male offspring given his mother’s genotype.

Let the relative risk for the offspring of a mother carrying one (two) copies of the variant allele be $S_{1} (S_{2})$ relative to the offspring of a mother carrying no copies. Then, $W_{G}=\left[ R_{0G}, R_{0G}S_{1},R_{0G}S_{2} \right]^{T}$, where the superscript “T” denotes transpose and $R_{0G}$ is the risk for a female child whose mother carried no copies of the variant. Similarly, $W_{B}=\left[ R_{0B}, R_{0B}S_{1},R_{0B}S_{2} \right]^{T}$, where $R_{0B}$is the risk for a male child whose mother carried no copies of the variant.

Consider the calculation of $P_{MM|D_{c}}$. Because the grandmother’s genotype is not risk-relevant, $P_{MM|D_{c}}=P_{M|D_{c}}V$. Because $\Pr\left[ D_{C} | M,C \right]\neq\Pr\left[ D_{C} | C \right]$ for maternal effects, Result 2 implies that we must calculate $P_{M|D_{c}}$ directly from the joint conditional distribution $\Pr\left[ M,F,C | D_{C} \right]$ (Table S2). For $K=p^{2}S_{2}+2p\left( 1-p \right)S_{1}+{(1-p)}^{2}$,

$$P_{M|D_{c}}=\left[ {(1-p)}^{2}K^{-1}, 2p\left( 1-p \right)S_{1}K^{-1}, p^{2}S_{2}K^{-1} \right],$$

enabling calculation of $P_{MM|D_{c}}$. Thus, the risk for a mother of an affected child is: $\Pr\left[ D_{M} | D_{C} \right]=P_{MM|D_{c}}W_{G}=\left( P_{M|D_{c}}V \right)W_{G}$.

To calculate $P_{FM|D_{c}}$, we proceed similarly. Under maternal effects, the conditional independence assumption $\Pr\left[ D_{C} | FM,F \right]=\Pr\left[ D_{C} | F \right]$ needed to apply Result 2 holds because, for maternal effects alone, genotypes from the father’s side of the family are not risk-relevant. Thus, $P_{FM|D_{c}}=P_{F|D_{c}}V$. In fact, $P_{F|D_{c}}=P_{\mathrm{HWE}}$ where $P_{\mathrm{HWE}}=\left[ \left( 1-p \right)^{2}, 2p\left( 1-p \right), p^{2} \right]$ is the Hardy-Weinberg equilibrium genotype distribution (Table S2). (Note that for maternal effects the father’s genotype distribution is not enriched for the risk allele by conditioning on the offspring’s disease status.) Consequently, $P_{FM|D_{c}}=P_{F|D_{c}}V=P_{\mathrm{HWE}}V=P_{\mathrm{HWE}}$. Thus, the risk for the father of an affected child is: $\Pr\left[ D_{F} | D_{C} \right]=P_{FM|D_{c}}W_{B}=P_{\mathrm{HWE}}W_{B}$.

Our assumptions about the population imply that: $\Pr\left[ D_{G} \right]=P_{\mathrm{HWE}}W_{G}$ and that $\Pr\left[ D_{B} \right]=P_{\mathrm{HWE}}W_{B}$.

Substituting these various results into C1 yields:

$$\mathbb{P=}\frac{{\Pr\left[ D_{M} | D_{C} \right]}/{\Pr\left[ D_{G} \right]}}{{\Pr\left[ D_{F} | D_{C} \right]}/{\Pr\left[ D_{B} \right]}}=\frac{\left( P_{MM|D_{c}}W_{G} \right)/\left( P_{\mathrm{HWE}}W_{G} \right)}{\left( P_{FM|D_{c}}W_{B} \right)/\left( P_{\mathrm{HWE}}W_{B} \right)}=\frac{\left[ \left( P_{M|D_{c}}V \right)W_{G} \right]/\left( P_{\mathrm{HWE}}W_{G} \right)}{\left( P_{\mathrm{HWE}}W_{B} \right)/\left( P_{\mathrm{HWE}}W_{B} \right)}$$

or

$$\mathbb{P=}\frac{\left( P_{M|D_{c}}V \right)W_{G}}{P_{\mathrm{HWE}}W_{G}}.$$

Under our assumptions and when genetic susceptibility depends on maternal genetic effects only, the inter-lineage parent relative risk is the same whether the risk for males and risk for females are the same or not.

**Grandparents**

From S1, the inter-lineage grandmother relative risk is:

$\mathbb{G=}\frac{\Pr\left[ D_{MM} | D_{C} \right]}{\Pr\left[ D_{FM} | D_{C} \right]}.$ (C2)

Under maternal effects, risk-relevant genotype for disease in the grandmother would be her mother. Thus, $\Pr\left[ D_{\mathrm{MM}} | {MMM=m,D}_{C} \right]=\Pr\left[ D_{\mathrm{MM}} | \mathrm{MMM}=m \right]$ for $m=0,1,2$ so that $\Pr\left[ D_{\mathrm{MM}} | D_{C} \right]=\sum_{m} \Pr\left[ MMM=m | D_{C} \right]\Pr\left[ D_{\mathrm{MM}} | \mathrm{MMM}=m \right]=P_{MMM|D_{c}}W_{G}$ where $P_{MMM|D_{c}}$denotes a 1×3 genotype probability vector containing $\Pr\left[ MMM=m | D_{C} \right]$ for $m=0,1,2.$ Since the great grandmother is not risk relevant for the disease in the great grandchild, $\Pr\left[ D_{C} | MMM,MM \right]=\Pr\left[ D_{C} | MM \right]$ and,

$$P_{MMM|D_{c}}=P_{MM|D_{c}}V=P_{M|D_{c}}V^{2}.$$

Similar calculations apply for $P_{FMM|D_{c}}$, however, because $P_{FM|D_{c}}=P_{\mathrm{HWE}}$, $P_{FMM|D_{c}}=P_{\mathrm{HWE}}$ too. When the paternal genome has no influence on risk, selection of families based on an affected child does not differentially select paternal progenitors’ genotypes.

Now $\Pr\left[ D_{\mathrm{MM}} | D_{C} \right]=P_{MMM|D_{c}}W_{G}=\left( P_{M|D_{c}}V^{2} \right)W_{G}$. Similarly, $\Pr\left[ D_{\mathrm{FM}} | D_{C} \right]=P_{FMM|D_{c}}W_{G}=P_{\mathrm{HWE}}W_{G}$. Substituting these results into C2 yields:

$$\mathbb{G=}\frac{\Pr\left[ D_{MM} | D_{C} \right]}{\Pr\left[ D_{FM} | D_{C} \right]}=\frac{P_{MMM|D_{c}}W_{G}}{P_{FMM|D_{c}}W_{G}}=\frac{\left( P_{M|D_{c}}V^{2} \right)W_{G}}{P_{\mathrm{HWE}}W_{G}}.$$
