# Supplementary material for: Asymmetry in Family History Implicates Nonstandard Genetic Mechanisms: Application to the Genetics of Breast Cancer
Source: PLoS Genet. 2014 Mar 20;10(3):e1004174. doi: 10.1371/journal.pgen.1004174 (PMC3961172; doi:10.1371/journal.pgen.1004174)
Supplement: Text S4 — Relationship between parent and grandparent asymmetry induced by a maternal effect. (DOCX) [file pgen.1004174.s007.docx]

**Text S4. Relationship between parent and grandparent asymmetry induced by a maternal effect.**

Denote the parental relative risk (mother vs. father) by $\mathbb{P}$ and the grandmothers’ relative risk (maternal vs. paternal) by $\mathbb{G}$. Under the simplifying assumptions of the manuscript (random mating, Mendelian inheritance, and HWE at the locus under study) and the specific assumptions that the only influence of a gene on an offspring’s risk is a maternally mediated effect and that $W_{G}=W_{B}$, these two relative risks are related by $\mathbb{G=}\left( 1+\mathbb{P} \right)/2$, or equivalently, $2\mathbb{G-P=}1$.

To prove that this relationship is correct in our simplified setting, start from the following matrix expressions for the relative risks (notation and derivation in S3) derived under the assumptions stated earlier: $\mathbb{P=}\frac{\left( P_{M|D_{c}}V \right)W_{G}}{P_{\mathrm{HWE}}W_{B}}$ and $\mathbb{G=}\frac{\left( P_{M|D_{c}}V^{2} \right)W_{G}}{P_{\mathrm{HWE}}W_{G}}$. Substituting these expressions into $2\mathbb{G-P=}1$ yields:

$$2\frac{\left( P_{M|D_{c}}V^{2} \right)W_{G}}{P_{\mathrm{HWE}}W_{G}}-\frac{\left( P_{M|D_{c}}V \right)W_{G}}{P_{\mathrm{HWE}}W_{B}}=1.$$

We multiply both sides by $P_{\mathrm{HWE}}W_{G}$, which is equal to $P_{\mathrm{HWE}}W_{B}$ by assumption, and factor the left side of resulting equation to get

$P_{M|D_{c}}\left( 2V^{2}-V \right)W_{G}=P_{\mathrm{HWE}}W_{G}.$ (D1)

For the matrix $V$ as defined in the manuscript, carrying out the matrix algebraic calculations reveals that

$$2V^{2}-V=\left[ \begin{matrix} \left( 1-p \right)^{2} & 2p\left( 1-p \right) & p^{2} \\ \left( 1-p \right)^{2} & 2p\left( 1-p \right) & p^{2} \\ \left( 1-p \right)^{2} & 2p\left( 1-p \right) & p^{2} \end{matrix} \right],$$

that is, each row of the matrix $2V^{2}-V$ is identical and equal to the row vector $P_{\mathrm{HWE}}$. Because $P_{M|D_{c}}$ is a probability vector that sums to 1, pre-multiplying the matrix $2V^{2}-V$ by the row vector $P_{M|D_{c}}$ yields a row vector that is a weighted average of the rows of the matrix $2V^{2}-V$. Because those rows are all the same, the weighted average is just one of the rows, that is, $P_{M|D_{c}}\left( 2V^{2}-V \right)=$ $P_{\mathrm{HWE}}$. Substituting this result into D1 proves that $2\mathbb{G-P=}1$ for any risk models $W_{G}$ and $W_{B}$ that embody purely maternal genetic effects.
