# Supplementary material for: Asymmetry in Family History Implicates Nonstandard Genetic Mechanisms: Application to the Genetics of Breast Cancer
Source: PLoS Genet. 2014 Mar 20;10(3):e1004174. doi: 10.1371/journal.pgen.1004174 (PMC3961172; doi:10.1371/journal.pgen.1004174)
Supplement: Text S5 — Asymmetry produced by an imprinted genetic variant. (DOCX) [file pgen.1004174.s008.docx]

**Text S5: Asymmetry produced by an imprinted genetic variant.**

We make the simplifying assumption that only the maternally-inherited copy is expressed, and denote the relative risk for a maternally inherited copy as *I*. Again we consider the grandmother’s relative risk $\frac{P_{MM|D_{c}}W_{G}}{P_{FM|D_{c}}W_{G}}$ except that now $W_{G}=\left[ R_{0G},\frac{R_{0G}\left( I+1 \right)}{2},R_{0G}I \right]^{T}$ for the parent-of-origin model. As with maternal effects, one calculates $P_{MM|D_{c}}$, the genotype distribution among maternal grandmothers of an affected child, by post-multiplying $P_{M|D_{c}}$, the genotype distribution among mothers of an affected child, by the matrix $V$, thus $P_{MM|D_{c}}=P_{M|D_{c}}V$. For $Q=1-p+Ip$, $P_{M|D_{c}}=$

$\left[ {(1-p)}^{2}Q^{-1}, p\left( 1-p \right)(I+1)Q^{-1}, p^{2}IQ^{-1} \right]$ (Table S3). A similar calculation, $P_{FM|D_{c}}=P_{F|D_{c}}V$, applies to paternal grandmothers; however, $P_{F|D_{c}}$is the HWE distribution, $P_{\mathrm{HWE}}$ (Table S3). Under the assumption that *I* is the same in both sexes, the grandfather’s relative risk, $\frac{P_{MF|D_{c}}W_{B}}{P_{FF|D_{c}}W_{B}}$ will be the same as the grandmother’s relative risk.
