# Supplementary material for: Asymmetry in Family History Implicates Nonstandard Genetic Mechanisms: Application to the Genetics of Breast Cancer
Source: PLoS Genet. 2014 Mar 20;10(3):e1004174. doi: 10.1371/journal.pgen.1004174 (PMC3961172; doi:10.1371/journal.pgen.1004174)
Supplement: Text S6 — Relationship between parental asymmetry induced by maternal effects and grandparental asymmetry induced by a parent-of-origin effect. (DOCX) [file pgen.1004174.s009.docx]

**Text S6: Relationship between parental asymmetry induced by maternal effects and grandparental asymmetry induced by a parent-of-origin effect.**

Denote the parental relative risk induced by maternal effects (mother vs. father) by $\mathbb{P}^{M}$ and the grandmothers’ relative risk induced by a parent-of-origin effect (maternal vs. paternal) by $\mathbb{G}^{I}$. We use the simplifying assumptions of the manuscript (random mating, Mendelian inheritance, and HWE at the locus under study). In addition, for maternal effects, we assume that the mode of inheritance is log-additive ($S_{2}=S_{1}^{2}$) and that the relative risk parameter (denoted $S=S_{1}$) and baseline risk (denoted $R_{0}$) are the same for males and females. For parent-of-origin effects, we also assume that the risk parameters (denoted $I$ and $R_{0}$) are the same for males and females. Under these assumptions, these two relative risks are related by $4\mathbb{G}^{I}-3=\mathbb{P}^{M}$ when $S=I$. To demonstrate this relationship, we start from the following matrix expressions for the relative risks: $\mathbb{P}^{M}=\frac{\left( P_{M|D_{c}}^{M}V \right)W^{M}}{P_{\mathrm{HWE}}W^{M}}$ and $\mathbb{G}^{I}=\frac{\left( P_{M|D_{c}}^{I}V \right)W^{I}}{P_{\mathrm{HWE}}W^{I}}$. The basic notation is defined in Appendices C and E; however, we use superscripts to distinguish maternal- or imprinting-related quantities and suppress sex-specific subscripts on $W^{M}$ and $W^{I}$ as we assume no sex differences in risk parameters.

Start with the calculation of $\mathbb{P}^{M}$. For compact notation, define $q=1-p$ where $p$ denotes the minor allele frequency. From S3, specialized to the log-additive risk model, we have that $P_{M|D_{c}}^{M}=K^{-1}\left[ q^{2}, 2pqS, p^{2}S^{2} \right]$ for $K={q^{2}+2pqS+p}^{2}S^{2}={(q+pS)}^{2}$, and that $W^{M}=R_{0}\left[ 1, S, S^{2} \right]^{T}$.

To derive the numerator of $\mathbb{P}^{M}$, first carry out a matrix multiplication to get the row vector $P_{M|D_{c}}^{M}V=K^{-1}\left[ q^{3}+pq^{2}S, pq^{2}+pqS+p^{2}qS^{2}, p^{2}qS+p^{3}S^{2} \right]$. Next, multiply that row vector by $W^{M}$ to get the scalar:

$\left( P_{M|D_{c}}^{M}V \right)W^{M}=R_{0}K^{-1}\left[ \left( q^{3}+pq^{2}S \right)+\left( pq^{2}S+pqS^{2}+p^{2}qS^{3} \right)+\left( p^{2}qS^{3}+p^{3}S^{4} \right) \right]$.

Noting that $p+q=1$, we have that $pqS^{2}=pq\left( p+q \right)S^{2}=p^{2}qS^{2}+ pq^{2}S^{2}$. Substituting this expression for $pqS^{2}$ into the expression for the numerator, collecting like terms, and re-arranging yields:

$$\left( P_{M|D_{c}}^{M}V \right)W^{M}=R_{0}K^{-1}\left[ \left( q^{3}+2pq^{2}S+p^{2}qS^{2} \right)+\left( pq^{2}S^{2}+2p^{2}qS^{3}+p^{3}S^{4} \right) \right]$$

$$=R_{0}K^{-1}\left[ q{(q+pS)}^{2}+pS^{2}{(q+pS)}^{2} \right]=R_{0}K^{-1}\left( qK+pS^{2}K \right)$$

$$=R_{0}\left( q+pS^{2} \right)$$

The denominator of $\mathbb{P}^{M}$ is: $P_{\mathrm{HWE}}W^{M}$=$R_{0}{(q+pS)}^{2}.$ Taking the quotient of numerator and denominator we have: $\mathbb{P}^{M}=\frac{R_{0}\left( q+pS^{2} \right)}{R_{0}{(q+pS)}^{2}}=\frac{q+pS^{2}}{{(q+pS)}^{2}}$.

Turn next to the calculation of $\mathbb{G}^{I}$. Adapting expressions from S5, we have $P_{M|D_{c}}^{I}=Q^{-1}\left[ q^{2}, pq\left( I+1 \right), p^{2}I \right]$ for $Q=q+pI$. Also, $W^{I}=R_{0}\left[ 1, \frac{I+1}{2}, I \right]^{T}$.

To derive the numerator of $\mathbb{G}^{I}$, first carry out a matrix multiplication to get the row vector $P_{M|D_{c}}^{I}V=Q^{-1}\left[ q^{3}+pq^{2}\left( \frac{I+1}{2} \right), pq^{2}+pq\left( \frac{I+1}{2} \right)+p^{2}qI, p^{2}q\left( \frac{I+1}{2} \right)+p^{3}I \right]$. Next, multiply that row vector by $W^{I}$ to get the scalar:

$$\left( P_{M|D_{c}}^{I}V \right)W^{I}=R_{0}Q^{-1}\times$$

$\left[ \left( q^{3}+pq^{2}\left( \frac{I+1}{2} \right) \right)+\left( pq^{2}\left( \frac{I+1}{2} \right)+pq\left( \frac{I+1}{2} \right)^{2}+p^{2}qI\left( \frac{I+1}{2} \right) \right)+\left( p^{2}qI\left( \frac{I+1}{2} \right)+p^{3}I^{2} \right) \right]$.

Again using $p+q=1$, $pq\left( \frac{I+1}{2} \right)^{2}=pq\left( p+q \right)\left( \frac{I+1}{2} \right)^{2}=p^{2}q\left( \frac{I+1}{2} \right)^{2}+ pq^{2}\left( \frac{I+1}{2} \right)^{2}$. Substituting this expression for $pq\left( \frac{I+1}{2} \right)^{2}$ into the expression for the numerator, collecting like terms, and re-arranging yields:

$$\left( P_{M|D_{c}}^{I}V \right)W^{I}=R_{0}Q^{-1}\times$$

$$\left\{ \left[ q^{3}+2pq^{2}\left( \frac{I+1}{2} \right)+p^{2}q\left( \frac{I+1}{2} \right)^{2} \right]+\left[ pq^{2}\left( \frac{I+1}{2} \right)^{2}+2p^{2}qI\left( \frac{I+1}{2} \right)+p^{3}I^{2} \right] \right\}$$

$=R_{0}K^{-1}\left\{ q\left[ q+p\left( \frac{I+1}{2} \right) \right]^{2}+p\left[ q\left( \frac{I+1}{2} \right)+pI \right]^{2} \right\}$.

Now, $q+p\left( \frac{I+1}{2} \right)=q+½pI+½p=½\left[ \left( q+pI \right)+(q+p) \right]=½\left( Q+1 \right)$. Similarly, $q\left( \frac{I+1}{2} \right)+pI=½\left[ \left( q+pI \right)+(q+p)I \right]=½\left( Q+I \right)$. Substituting these expressions into the numerator yields:

$\left( P_{M|D_{c}}^{I}V \right)W^{I}=R_{0}Q^{-1}\left[ \frac{q}{4}\left( Q+1 \right)^{2}+\frac{p}{4}\left( Q+I \right)^{2} \right]$.

Expanding the right-hand side and collecting terms in powers of $Q$ leads to:

$$\left( P_{M|D_{c}}^{I}V \right)W^{I}=\frac{R_{0}Q^{-1}}{4}\left[ Q^{2}\left( p+q \right)+2Q\left( q+pI \right)+\left( q+pI^{2} \right) \right]$$

$=\frac{R_{0}Q^{-1}}{4}\left[ {3Q}^{2}+\left( q+pI^{2} \right) \right]=\frac{R_{0}}{4}\left[ 3Q+\frac{q+pI^{2}}{Q} \right]$.

The denominator of $\mathbb{G}^{I}$ is:

$$P_{\mathrm{HWE}}W^{I}=R_{0}\left[ q^{2}+pq\left( I+1 \right)+p^{2}I \right]=R_{0}\left[ q\left( p+q \right)+pI\left( p+q \right) \right]$$

$=R_{0}\left( q+pI \right)=R_{0}Q$.

Taking the quotient of numerator and denominator gives:

$\mathbb{G}^{I}=\frac{R_{0}}{4}\left[ 3Q+\frac{q+pI^{2}}{Q} \right]\left[ R_{0}Q \right]^{-1}=\frac{3}{4}+\frac{q+pI^{2}}{4Q^{2}}=\frac{3}{4}+\frac{q+pI^{2}}{4\left( q+pI \right)^{2}}$.

Using this expression for $\mathbb{G}^{I}$,

$4\mathbb{G}^{I}-3=4\left[ \frac{3}{4}+\frac{q+pI^{2}}{4\left( q+pI \right)^{2}} \right]-3=\frac{q+pI^{2}}{\left( q+pI \right)^{2}}$.

Replacing $I$ with $S$ in the rightmost expression reveals that, under the stated assumptions, $4\mathbb{G}^{I}-3=\mathbb{P}^{M}$as claimed.
